# Temporal trends in LDL cholesterol levels from 2009 to 2023: a comprehensive analysis of 2.4 million patient records in São Paulo, Brazil

**DOI:** 10.31744/einstein_journal/2026AO1104

**Published:** 2026-06-23

**Authors:** Paulo Roberto Telles Pires Dias, Queoma Silveira Mariante, Flavia Paiva Proença Lobo Lopes

**Affiliations:** 1 Instituto de Ensino e Pesquisa Diagnosticos da America SA Barueri SP Brazil Instituto de Ensino e Pesquisa, Diagnosticos da America SA, Barueri, SP, Brazil.; 2 Universidade do Estado do Rio de Janeiro Rio de Janeiro RJ Brazil Universidade do Estado do Rio de Janeiro, Rio de Janeiro, RJ, Brazil.; 3 Universidade Federal Fluminense Niterói RJ Brazil Universidade Federal Fluminense, Niterói, RJ, Brazil.; 4 Universidade Federal do Rio de Janeiro Rio de Janeiro RJ Brazil Universidade Federal do Rio de Janeiro, Rio de Janeiro, RJ, Brazil.

**Keywords:** Cholesterol, LDL, Cardiovascular diseases, Primary prevention

## Abstract

**Objective:**

This study aimed to evaluate monthly trends in low-density lipoprotein cholesterol (LDL-C) levels in the examined population.

**Methods:**

Monthly trends in LDL-C levels from 2009 to 2023 were analyzed in a predominantly outpatient cohort that underwent testing at *Laboratório Dasa* units in São Paulo, Brazil. A comprehensive analysis was conducted using 2,469,979 complete records with valid LDL-C results obtained from databases of a national clinical laboratory. Monthly mean and median LDL-C values were calculated and analyzed according to sex and age groups.

**Results:**

Piecewise regression analysis of temporal trends identified a breakpoint in 2018, indicating a change in slope. The average monthly decline in LDL-C levels from 2009 to 2018 was 0.159 mg/dL (p<0.001). After 2018, the average LDL-C level increased, with a monthly increase of 0.142 mg/dL (p<0.001). This pattern was consistent across both sexes and across most of the examined age groups. Autoregressive integrated moving average time series analysis revealed seasonal variations in LDL-C levels, with a tendency towards lower levels during warmer months and higher levels during colder months.

**Conclusion:**

Model projections suggest a potential upward trend in LDL-C levels in the coming years. These trends indicate that the historical decline in LDL-C levels observed from 2009 to 2018 has not persisted in recent years. Further research is needed to elucidate the underlying causes of these observed trends and to develop strategies aimed at reducing serum lipid levels associated with cardiovascular risk.

## In Brief

This study analyzed 2.4 million patient records from São Paulo, Brazil, from 2009 to 2023 and identified a significant downward trend in total and low-density lipoprotein cholesterol levels across all age groups and sexes. These findings suggest improved lipid control and highlight the impact of evolving cardiovascular prevention strategies in one of the world's largest urban centers.

## Highlights

■ This large real-world study analyzed blood cholesterol trends over a 15-year period in São Paulo, Brazil.
■ Mean total and LDL-C decreased significantly across all groups.
■ A higher proportion of patients achieved recommended LDL-C target levels.
■ The observed trends may reflect improvements in cardiovascular prevention strategies and treatment efficacy.

## Graphical abstract

**Figure:**
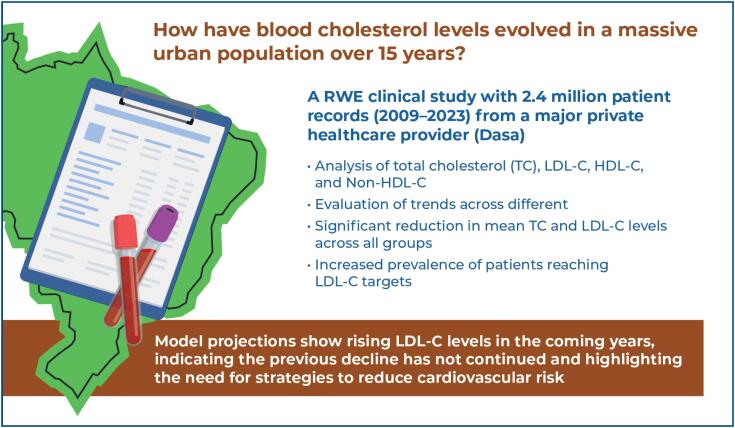

## INTRODUCTION

Serum lipids are pillars of proper body function. These molecules, including total cholesterol, low-density lipoprotein cholesterol (LDL-C), high-density lipoprotein cholesterol (HDL-C), and triglycerides, are crucial for various biological processes, including maintenance of cell membrane structure, hormone production, and the absorption of fat-soluble vitamins.

Among these lipids, LDL-C levels are strongly associated with the risk of cardiovascular disease (CVD) and coronary artery disease (CAD).^(1-3)^ Reduction of LDL-C levels can slow the progression of atherosclerotic plaque and decrease the incidence of atherosclerotic cardiovascular disease (ASCVD). A meta-analysis demonstrated that major vascular events such as acute myocardial infarction, CAD, death, stroke, and coronary revascularization were reduced by 22% over 5 years for every 1 mmol/L reduction in LDL-C.^(4,5)^

In this context, understanding the trends in serum LDL-C levels within the population plays an important role in promoting public health. The increasing availability of large-scale laboratory databases, together with the use of robust statistical tools, has enabled comprehensive analyses of temporal lipid trends.^(6)^ Temporal trends may reflect changes in dietary habits, lifestyle, genetic factors, medical interventions, and health policies. Monitoring and analyzing these trends can help identify potential intervention targets for preventing CVDs, improving quality of life, and reducing healthcare costs associated with lipid-related conditions.^(7)^ Recent studies conducted in the United States, Europe, and Asia^(8-11)^ have analyzed LDL-C trends over time, revealing regional and temporal variations linked to public health measures, lifestyle changes, and pharmacological interventions. These studies provide a valuable context for examining LDL-C trends in Brazil, where such investigations remain limited.

## OBJECTIVE

This study aimed to describe the LDL-C profile, evaluate temporal trends in LDL-C levels, and examine possible associations with available demographic variables.

## METHODS

### Study design

This was a descriptive, retrospective, non-interventional study. As this study was retrospective in nature, predefined exclusion criteria and clinical admission parameters were not established, which may limit the generalizability and clinical applicability of the results. All tests were performed upon request by the attending physician as part of routine clinical care, without the influence of the research team.

### Data source

The primary data source was the centralized Dasa clinical laboratory database. The secondary data source was a project-specific anonymized extract covering the period from 2009 to 2023, created specifically for this study. Owing to privacy and data protection regulations under the Brazilian General Data Protection Law (*Lei Geral de Proteção de Dados*), the dataset is not publicly available but may be provided in an anonymized form upon reasonable request. Data extraction was performed using standardized protocols.

### Variables

All fields underwent routine validation procedures to ensure completeness, consistency, and accuracy. Only records containing valid and complete data on age, sex, examination date, and LDL-C values were included in the analysis.

### Statistical analysis

All analyses conducted to address the main, secondary, and exploratory objectives were either descriptive or assessed for associations between variables available in the database. Continuous variables are presented with mean ± standard deviation (SD) and/or median with interquartile ranges (IQR), depending on the distribution of the data. Normally distributed data were compared using Student's *t*-test, whereas non-normally distributed continuous data were compared using the Mann-Whitney U test. Categorical variables are presented as percentages and were compared using the chi-square test. Autoregressive integrated moving average (ARIMA) and Piecewise regression models were used to study, decompose, and forecast the time series. A 95% confidence interval (alpha error, 5%) was adopted as the threshold for statistical significance. The demographic characteristics of the study participants (sex, age, and examination date) were analyzed quantitatively and qualitatively.

The statistical procedures applied to the data generated point estimates and corresponding confidence intervals for occurrence or effect measures. The data and results of the different comparisons are presented graphically. Data stability was assessed using statistical comparisons at different time intervals in the available database to evaluate whether the observed results remained consistent over time. All statistical analyses were performed using R statistical software (version 4.3.0).^(6)^ The packages used included ‘forecast’ for ARIMA modeling, ‘segmented’ for piecewise regression, and ‘ggplot2’ for graphical visualization.

This study was approved by the Research Ethics Committee of *Hospital Nove de Julho* (CAAE: 68047223.8.0000.5455; #6.536.965).

## RESULTS

A total of 2,469,979 patient records with complete and valid results were analyzed from the databases. The median age of the participants was 37 years, and 61.3% were female. The distribution of LDL-C levels according to sex and age is summarized in table 1. The mean age of participants was 38.12 (±18.8) years (38.4 [±18.3] years for females and 37.7 [±19.6] years for males). The annual number of examinations increased over the study period from 83,645 in the initial year to 111,742 in later years, reaching a maximum of 234,253 in 2021.

**Table 1:** Median LDL-C levels by sex and age group (2009-2023, pooled data)

Sex	Age group	n	Median LDL-C (mg/dL)	% within sex
Female	≤17	196,846	92.3	13.0
	18-39	651,673	102.0	43.1
	40-59	462,134	115.0	30.5
	≥60	202,473	110.0	13.4
Male	≤17	188,333	90.1	19.7
	18-39	317,614	111.0	33.2
	40-59	316,641	116.0	33.1
	≥60	134,265	102.0	14.0

The median LDL-C value for the entire sample was 103mg/dL (IQR=37.6). The median LDL-C value was 102.4 (IQR=35.7) for females and 104.2 (IQR=40.4) for males. A decline of approximately 4% in monthly median LDL-C levels was identified from 2009 to 2023, decreasing from 110.9 mg/dL in the first semester to 106.7 mg/dL in the final semester analyzed. Overall, 44% of individuals had desirable LDL-C levels (<100mg/dL), whereas the prevalence of dyslipidemia (≥130mg/dL) in the sample was 20.2%. Among adolescents (aged 12-17 years), who comprised 5.46% of the sample, the prevalence of dyslipidemia was 4.6%.

**Figure 1:**
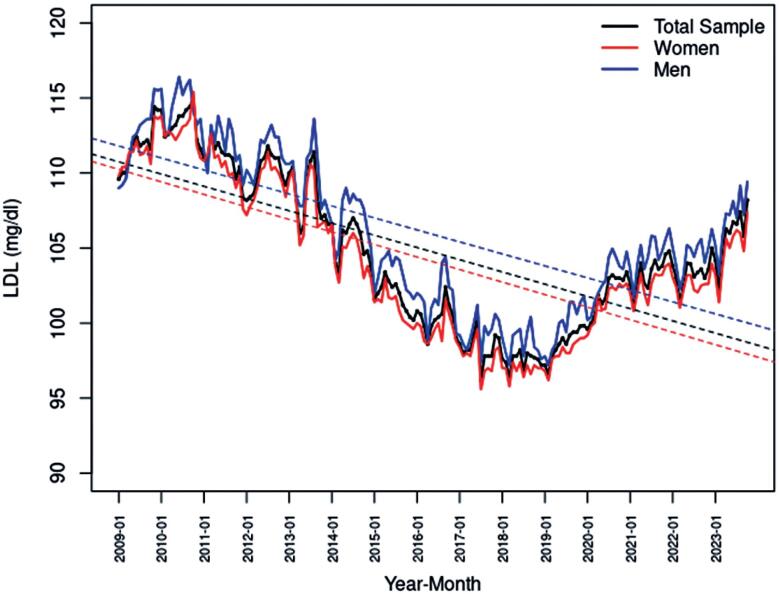
Time series plot of monthly median LDL-C levels in the study population, stratified by sex, from 2009 to 2023. Regression lines illustrate the trends for each group. The dataset used for this analysis included a total of 2,469,979 observations

Monthly median LDL-C values stratified by sex are shown in figure 1, along with their corresponding regression lines. In general, the trends were similar for both sexes, with LDL-C levels in males being consistently higher than those in females throughout the study period.

**Figure 2:**
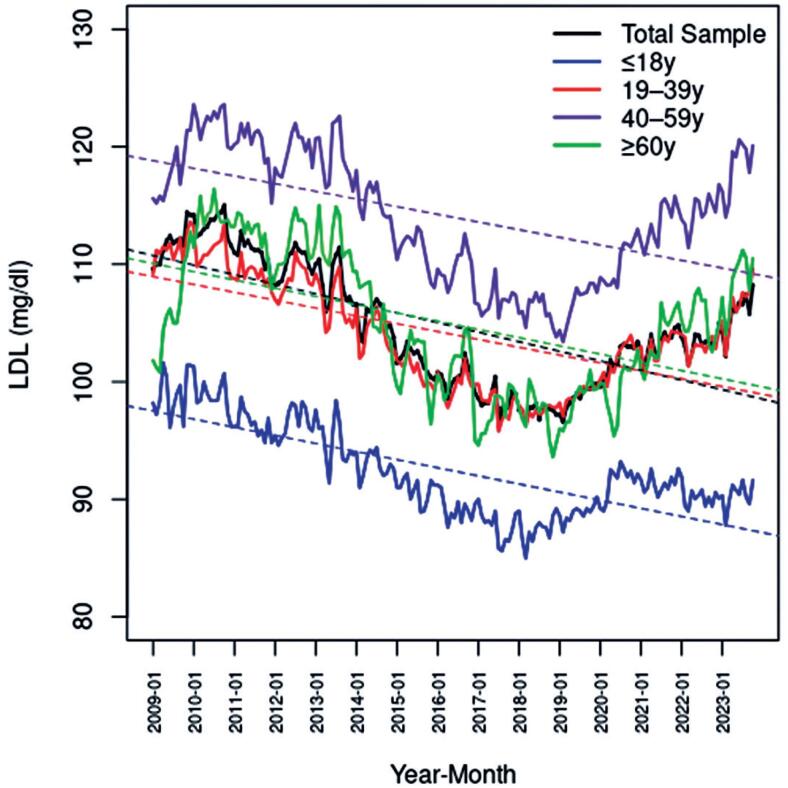
Time series plot of monthly median LDL-C levels in the study population overall and stratified by age group from 2009 to 2023. Regression lines illustrate the trends for each of the specified categories. The dataset used for this analysis included a total of 2,469,979 observations

In figure 2, the same type of graph is created for different age groups. Although the temporal trends were similar, more pronounced differences were observed in the median LDL-C levels between the analyzed categories. Higher levels were observed in the 40-59-year age group, whereas lower levels were observed in individuals aged ≤19 years. LDL-C levels generally increased with age, except in the oldest age group (>60 years), in which a reduction in LDL-C levels was observed. These differences were supported by statistical analyses, including ANOVA and post hoc comparisons across age groups (*p*<0.001).

**Figure 3:**
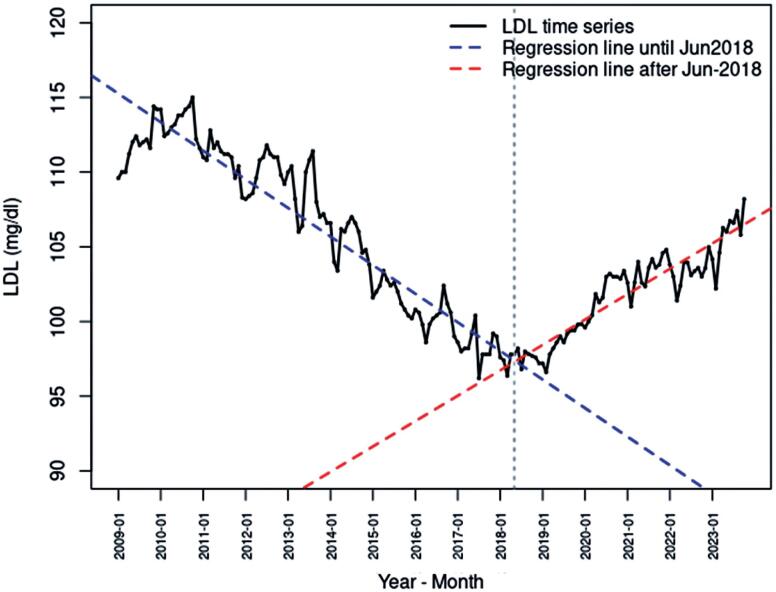
Time series plot of monthly median LDL-C levels in the segmented regression sample. Piecewise regression analysis of temporal trends indicates a breakpoint in 2018. The dataset included 2,469,979 observations

In the two graphs presented earlier, it a decline in the rates can be observed over the entire study period. However, distinct differences in trends are evident for the two periods: 2009-2018 and 2018-2023. While the first period (2009-2018) exhibited a consistent decline in the rates, the second period (2018-2023) exhibited an increasing trend. Thus, the two regression lines have divergent characteristics, as shown in figure 3. In the initial period up to 2018, time was a statistically significant predictor of decreasing LDL-C levels (β=-0.159, *p*<0.001, R2=0.90). In contrast, in the subsequent period, time was a statistically significant predictor of increasing LDL-C levels in the sample (β=0.142, *p*<0.001, R2=0.85). Considering the variation in median levels, a decrease of approximately 15.5% in the monthly LDL-C medians was observed from 2009 to 2018. Conversely, an increase of 9.5% was observed from 2018 to 2023. Piecewise regression analysis of the temporal trends identified the inflection point that occurred in June 2018 using segmented regression modeling.

**Figure 4:**
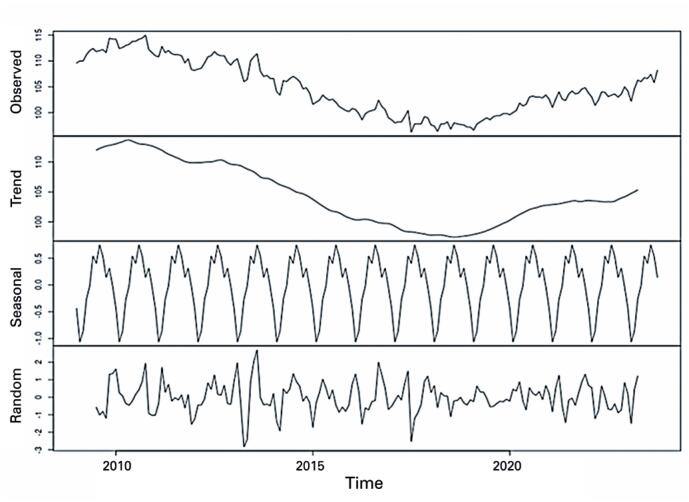
Additive decomposition graphs of the time series of monthly median LDL-C levels in the study population. The dataset included 2,469,979 observations

In addition to the variations described above, time series analysis using ARIMA models revealed seasonal fluctuations in LDL-C levels (Figure 4). Specifically, LDL-C levels tended to be lower during warmer months and higher during colder months. Forecasts generated using these models indicate a potential increasing trend in LDL-C levels in the coming years.

## DISCUSSION

This study contributes to existing scientific literature by demonstrating trends in LDL-C levels in a large sample of a mostly outpatient population. Approximately 2.5 million patients who underwent testing at Dasa laboratory units in São Paulo, Brazil, from 2009 to 2023 were included, making this, to our knowledge, the largest retrospective Brazilian study examining LDL-C trends to date. All LDL-C measurements were performed using standardized enzymatic methods according to the manufacturer's specifications and internal laboratory quality control procedures. The laboratory was accredited according to international and national standards (*e.g*., PALC/SBPC and ISO 15189), ensuring test reproducibility and analytical consistency throughout the study period.

In this study, we observed an approximately 8% decrease in the median monthly LDL-C level from 2009 to 2018. Theoretically, this reduction may reflect a significant improvement in public health promotion. Since each 10mg/dL reduction in LDL-C is associated with an approximately 5%-13% reduction in ASCVD events and mortality,^(7)^ the 15.5mg/dL reduction observed in our study could correspond to a 7.8%-20.1% reduction in CVD mortality, although this estimate does not account for changes in other risk factors over time.

A reversal in the decline in LDL-C levels was observed until 2018, followed by an increase in median LDL-C levels in subsequent years. This pattern was observed across all age groups (except for the youngest) and in both sexes. Other studies^(8-11)^ have reported similar declining trends in LDL-C levels; however, few studies extend beyond 2018, making direct comparison with the later years analyzed in this study challenging. Although comparisons with post-2018 international data would be valuable, access to harmonized patient-level datasets remains limited. Further studies may help address this gap.

Seasonal variations in LDL-C levels, characterized by a tendency towards lower levels during warmer months and higher levels during colder months, have been reported in several other studies,^(12-15)^ confirming the findings of the present study. The mechanisms underlying these seasonal variations remain uncertain, and whether specific diagnostic thresholds or guidelines should be adjusted to account for seasonal variations also remains unclear.^(16)^ Although the observed pattern aligns with the expected seasonal changes, no meteorological data were analyzed in this study to confirm the associations with temperature.

It is beyond the scope of the present study to determine the specific factors in medical practice or changes in patient behavior that may explain these findings, particularly the observed increase in LDL-C levels after 2018. Additionally, whether the COVID-19 pandemic contributed to the observed trend remains unclear. Notably, however, a previous study has reported increased levels of LDL-C during follow-up among patients who experienced severe COVID-19.^(17)^

The variation in LDL-C levels by age group observed in this study (lower levels in young individuals, higher levels in middle-aged adults, and lower levels in older adults) aligns with the results of a previous study.^(8)^ Other studies have also reported a decrease in cholesterol levels among older age groups.^(18-21)^ Some studies suggest that the high prevalence of health issues in older individuals is a significant determinant of the inverse association between age and cholesterol levels. Indicators of compromised health have been associated with a greater likelihood of hypocholesterolemia in both men and women.^(22)^ These findings suggest that achieving improvements in cardiovascular health may become increasingly challenging in the coming years if preventive measures are not taken and the recent upward trend in median levels persists.

Monthly median LDL-C levels were consistently higher in men than in women throughout the study period. This difference may reflect significant disparities between the sexes in the prescription and effectiveness of lipid-lowering interventions, including statins and lifestyle modifications. Additionally, over the entire study period, 44% of individuals had desirable LDL-C levels (<100mg/dL). Other studies, including national studies, have also reported higher LDL-C levels in men than in women,^(8,19,23)^ possibly indicating a higher cardiovascular risk among men. However, Brazilian studies based on data from a national health survey reported higher rates of hyperlipidemia among women.^(24,25)^

The prevalence of dyslipidemia (LDL-C ≥130mg/dL) in the analyzed sample was 20.2%, slightly higher than that reported in a national health survey (18.6%).^(24)^ However, these rates were lower than those found in similar studies conducted in the United States, where the prevalence decreased from 44.0% in 1999-2000 to 26.4% in 2017-2018.^(9)^ Among adolescents, the prevalence of LDL-C dyslipidemia in this study (4.6%) was also slightly higher than that reported in a national school-based study conducted between 2013 and 2014, which found prevalences of 3.7% among those aged 12-14 years and 3.4% among those aged 15-17 years.^(26)^ This difference may be explained by the sample variation between the studies, as the latter was school-based, whereas the present study included adolescents receiving medical prescriptions. Additionally, the proportion of individuals with desirable LDL-C levels (<100mg/dL) in our study was 44%, which is considerably higher than the prevalence reported in international population-based studies (23% and 26%, respectively).^(8,27)^

Despite these findings, this study has some limitations owing to its retrospective design. The study population comprised patients receiving care and undergoing physician-requested laboratory testing at Dasa laboratory units in São Paulo and may not fully reflect the general population of Brazil. Furthermore, clinical information, as well as details regarding lifestyle and medication use, were not collected. Given the retrospective nature of the study, the lack of predefined clinical inclusion or exclusion criteria limited control over potential confounding factors. Therefore, the findings should be interpreted as observational and exploratory, reflecting the patterns observed in a large outpatient-tested population rather than in the general Brazilian population. Although many findings from this study warrant further investigation to better clarify the underlying dynamics, the data suggest that after approximately 11 years of improvement in LDL-C levels, a decline occurred between 2019 and 2023.

## CONCLUSION

The observed differences in LDL-C levels across age groups and sexes highlight the need for targeted interventions and healthcare strategies. In addition, the prevalence of dyslipidemia in the study population, although slightly higher than the national average, underscores the ongoing public health challenge posed by cardiovascular risk factors.

Overall, this study highlights the dynamic nature of LDL-C trends and underscores the importance of continuous monitoring and intervention strategies to mitigate cardiovascular risk and promote public health. Further research is needed to better understand the determinants of these trends and develop targeted interventions aimed at reducing the burden of cardiovascular disease in the population.

## Data Availability

The data are not publicly available. Non-aggregated data cannot be shared because the Ethics Committee-approved protocol prohibits the disclosure of individual-level data to protect participants confidentiality.
